# Angioedema Secondary to tPA Use in Acute Ischemic Stroke Patient with Hypertension: A Case Report

**DOI:** 10.5811/cpcem.2021.1.49582

**Published:** 2021-04-23

**Authors:** Chris Kim, Andrea Hladik

**Affiliations:** Eisenhower Medical Center, Department of Emergency Medicine, Rancho Mirage, California

**Keywords:** tPa, angioedema, stroke

## Abstract

**Introduction:**

A well-documented complication of administering tissue plasminogen activator (tPA) in stroke patients is acute intracranial bleeding. A lesser known but still significant complication is angioedema secondary to tPA administration, which can develop in certain individuals with risk factors such as angiotensin converting enzyme (ACE) inhibitor use and location of the stroke. Knowing the potential for this life-threatening complication and being prepared for its proper management is vital for emergency physicians.

**Case Report:**

We report a 53-year-old Black female who presented to the emergency department with sudden onset of slurred speech and a facial droop. She was found to have an acute ischemic stroke and tPA was administered. She subsequently developed angioedema. Retrospectively, the patient was found to have risk factors that are thought to predispose patients to tPA-induced angioedema.

**Conclusion:**

Risk factors associated with angioedema secondary to tPA administration have been documented in patients taking ACE inhibitors, as well as patients who develop strokes in the frontal lobe. While many cases may be mild, some patients may develop life-threatening angioedema. Although this complication does not necessarily contraindicate tPA use, it is prudent for the emergency physician to be vigilant for its development, prepared for its treatment, and to be diligent in assessing the need for control of the patient’s airway.

## INTRODUCTION

The risk of intracranial hemorrhage is a known complication of tissue plasminogen activator (tPA) administration in stroke patients. Although reported rates vary, the risk is thought to be around 6%.^1^ Because of the devastating effects of this complication, multiple precautions and restrictions to the use of tPA have been outlined in approved guidelines set forth by the American Heart Association and the American Stroke Association.^2^ Multiple cases of tPA-associated angioedema have been documented in the literature with an estimated incidence of approximately 5%.^1^ Review of the published literature (including database searches in PubMed) shows discussion of the acute management of tPA-associated angioedema. Additionally, while the pathophysiologic mechanism for the development of angioedema in this circumstance has been proposed, it has not been confirmed. Angioedema secondary to tPA infusion can range from mild to severe and may require intubation for protection of the airway.^3^

Given that tPA-associated angioedema can be trivial or life-threatening, awareness and preparation should be discussed prior to administration of the drug. This case we report here helps highlight the known risk factors of tPA-associated angioedema, as well as the therapeutic interventions currently suggested for management of such cases. In addition, this case helps corroborate previously published literature regarding risk factors thought to contribute to the development of angioedema with tPA infusion.

## CASE REPORT

A 53-year old Black female presented to the emergency department (ED) with sudden onset of a facial droop and slurring of her words. The patient was last seen well 45 minutes prior to arrival. She had previously been seen three days earlier in the ED for hypertension and admitted to stopping her antihypertensives four months prior. She was prescribed antihypertensives at that time with recommendations to follow up with her primary care provider. A day prior to her presentation she had restarted lisinopril 20 milligrams (mg) daily, amlodipine 5 mg daily, and furosemide 20 mg daily as instructed by her primary care provider. Additional home medications included metformin 500 mg twice daily and potassium chloride.

The patient denied any headache, visual changes, dizziness, or numbness. She was found to be hypertensive upon arrival with blood pressure of 200/106 millimeters mercury (mm Hg), heart rate 130 beats per minute, respiratory rate 20 respirations per minute, pulse oximetry of 98% on room air, and temperature of 98.7° Fahrenheit. Initial neurologic exam showed the patient to be oriented to person, place, and time, with an obvious right-sided facial droop, deviation of tongue to the right side, and slurred speech. There were no initial motor deficits noted in her extremities as she was able to lift both her upper and lower extremities against gravity. Visual fields were grossly intact as was finger-to-nose testing. The remainder of her physical exam was otherwise unremarkable. Initial National Institutes of Health (NIH) stroke scale score was calculated to be approximately 2–3. The patient was taken for a computed tomography (CT) angiography of head and neck with and without contrast immediately after her initial exam.

The CT angiography of head and neck with and without contrast showed no acute intracranial findings, and no high-grade stenosis or occlusion in the head and neck through the internal carotid arteries. Vertebral artery imaging was obscured secondary to movement, and a thyroid goiter was also present. Upon return from CT, serial neurological exams were performed, which revealed right upper extremity weakness, thus increasing the NIH stroke scale score to 3–4. Tele-neurology was consulted and tPA was recommended. The patient and her husband agreed with the treatment plan regarding recommendations for tPA infusion. Tissue plasminogen activator was then administered for a total of 72.19 mg. A single dose of labetalol for 10 mg intravenous (IV) was initiated for blood pressure control.

During tPA infusion, the patient developed swelling of the right upper lip and right side of the tongue. It was believed that the tPA was the cause of her angioedema, and she was subsequently given diphenhydramine 50 mg IV in addition to methylprednisolone 125 mg IV. The infusion of tPA had coincidentally finished as the patient first began to develop angioedema. As a result, there was no need to discontinue the infusion, which is usually recommended when angioedema occurs. The patient remained hemodynamically stable, was able to maintain her airway, and did not require intubation while observed in the ED. She was subsequently admitted to the intensive care unit with the diagnosis of acute ischemic stroke.

**CPC-EM Capsule**

What do we already know about this clinical entity?

*Angioedema secondary to tissue-plasminogen activator (tPA) use is a well-documented occurrence in patients presenting with an acute ischemic stroke*

What makes this presentation of disease reportable?

*This case report corroborates the risk factors associated with angioedema secondary to (tPA) use as well as current recommendations for management of this condition*.

What is the major learning point?

*The major learning point is to recognize the condition and to take steps to ensure airway protection and resolution of the condition, should it arise*.

How might this improve emergency medicine practice?

*This case report highlights risk factors that can lead to this condition to allow physicians to readily recognize this condition and appropriately manage it with current guidelines*.

Prior to leaving the ED, serial neurological exams revealed improvement in her right upper extremity weakness, but she continued to have lingering facial droop. Reassessment of the patient’s angioedema showed improvement with only minimal swelling of the right upper lip and right side of tongue. She continued to maintain a patent airway. Initial laboratory studies included complete blood count, electrolyte panel, and prothrombin time/international normalized ratio and returned normal, except for mild hypokalemia of 3.1 millimoles per liter (mmol/L) (reference range 3.5–5.0 mmol/L) and elevated glucose of 226 (60–126 mg/dL).

After admission to the hospital, the patient underwent magnetic resonance imaging, which identified a subacute evolving infarct in the posterior left frontal lobe with a possible additional area of evolving infarct in the right frontal lobe as well. Magnetic resonance angiography revealed no primary vascular lesion such as an aneurysm, arteriovenous malformation, or arterial narrowing. During her hospital course, she continued to show improvement in her right upper extremity weakness and some improvement in the facial droop. Continued observation showed improvement in the angioedema over the course of a couple of days as well.

## DISCUSSION

Angioedema occurs due to an increase in local vascular permeability in submucosal or subcutaneous tissue. Histamine and bradykinin are vasoactive mediators usually involved in the development of angioedema, with most cases being mediated by one or the other.^4^ Some investigators indicate that both may be involved in certain cases.^5^ The use of tPA leads to fibrinolysis by hydrolyzing plasminogen to plasmin. Plasmin may play a role in the development of angioedema by activating the kinin pathway, which in turn leads to the formation of bradykinin. Plasmin also activates the complement system, leading to the eventual degranulation of mast cells and the release of histamine,^3^,^6^ which results in the formation of tissue swelling.

Angioedema after tPA infusion is a known complication and has an incidence of approximately 1–5%.^7^ Additionally, it is suggested that angioedema will typically occur on the contralateral side of the ischemic hemisphere.^6^ Several studies have proposed certain predisposing factors to the development of angioedema with the administration of tPA. A study performed by Hurford et al. identified that the use of angiotensin converting enzyme (ACE) inhibitors increases the risk of angioedema post-tPA use.^8^ Another study performed by Myslimi et al. suggests that when patients with hypertension are treated with ACE inhibitors and receive tPA, the rate of angioedema can occur as frequently as 1 in 6 patients.^9^ Fröhlich et al. suggests that angioedema secondary to tPA in patients with ischemic stroke in the insulo-opercular region is due to bradykinin effects causing vasodilation and increasing vascular permeability as described above.^10^ Other studies have also suggested that acute stroke in the frontal and insular cortex of the brain increase s the risk of angioedema post-tPA use.^6^ The rate of angioedema in acute ischemic stroke cases involving a total insular infarct is suggested to occur in about 1 in 10 patients.^9^

These findings support and align with the presentation of our patient. Prior to her arrival to the ED, she was restarted on ACE inhibitors to manage her hypertension. She subsequently presented with an ischemic stroke to the left frontal lobe with a resulting hemi-orolingual angioedema on the right side after receiving tPA. The use of ACE inhibitors as well as the development of a left frontal lobe stroke are likely to have increased her risk of developing hemi-orolingual angioedema and is supported by the above findings.

As previously mentioned, angioedema associated with the use of tPA is typically mild, although there have been severe cases leading to airway compromise as well as death.^3^,^11^ As a result of this risk, caution must be taken to monitor the patient’s airway to avoid any respiratory compromise. Management should include protection of the airway, discontinuation of the tPA-infusion if still in process, holding any ACE inhibitors, and the rapid administration of methylprednisolone 125 mg, diphenhydramine 50 mg, and ranitidine 50 mg or famotidine 20 mg. If the angioedema does not resolve, 0.1% epinephrine in 0.3 milliliters (mL) as a intramuscular injection or 0.5mL nebulizer treatment can also be administered. In cases of refractory angioedema, it has been suggested that complement component (C1)-esterase inhibitors can be considered as an additional therapeutic measure.^12^,^13^

For our patient, these medications were chosen based on recommendations and guidelines for tPA-associated angioedema on UpToDate (Wolters Kluwer Health, Waltham, MA). There is questionable efficacy of these medications for the treatment of tPA-associated angioedema (and angioedema in general); these medications seem to have varying to limited degrees of success with administration. This may be due to the kallikrein-bradykinin pathway that can lead to angioedema and that corticosteroids and antihistamines do not directly act on bradykinin. Other agents described by Powers et al. include C1-esterase inhibitors. However, these agents have unproven efficacy in current literature and should be used with prudent judgment.

## CONCLUSION

This case report exemplifies the typical patient possessing the possible risk factors associated with the development of angioedema secondary to use of tPA in the treatment of acute stroke. Our patient was on an ACE inhibitor and she was shown to have developed an infarct in the frontal lobe. Prompt management of stroke patients is key for patient outcomes, and being aware of all the complications associated with the use of tPA is vital to properly managing these patients. Fortunately, our patient did not develop life-threatening angioedema. However, she was certainly at risk given the clinical presentation and all factors considered. Tissue plasminogen activator does not necessarily need to be withheld in such a patient, but emergency physicians must be well acquainted with the possibility of this life-threatening complication in order to deal with it should the need arise.

## References

[1] Miller DJ, Simpson JR, Silver B (2011). Safety of thrombolysis in acute ischemic stroke: a review of complications, risk factors, and newer technologies. Neurohospitalist.

[2] Powers WJ, Rabinstein AA, Ackerson T (2019). Guidelines for the early management of patients with acute ischemic stroke: 2019 update to the 2018 Guidelines for the Early Management of Acute Ischemic Stroke: A Guideline for Healthcare Professionals From the American Heart Association/American Stroke Association. Stroke.

[3] Sczepanski M, Bozyk P (2018). Institutional incidence of severe tPA-Induced angioedema in ischemic cerebral vascular accidents. Crit Care Res Pract.

[4] Li HH (2018). Angioedema.

[5] Asero R, Bavbek S, Blanca M (2013). Clinical management of patients with a history of urticaria/angioedema induced by multiple NSAIDs: an expert panel review. Int Arch Allergy Immunol.

[6] Hill MD, Lye T, Moss H (2003). Hemi-orolingual angioedema and ACE inhibition after alteplase treatment of stroke. Neurology.

[7] Burd M, McPheeters C, Scherrer LA (2019). Orolingual angioedema after tissue plasminogen activator administration in patients taking angiotensin-converting enzyme inhibitors. Adv Emerg Nurs J.

[8] Hurford R, Rezvani S, Kreimei M (2015). Incidence, predictors and clinical characteristics of orolingual angio-oedema complicating thrombolysis with tissue plasminogen activator for ischaemic stroke. J Neurol Neurosurg Psychiatry.

[9] Myslimi F, Caparros F, Dequatre-Ponchelle N (2016). Orolingual angioedema during or after thrombolysis for cerebral ischemia. Stroke.

[10] Fröhlich K, Macha K, Gerner ST (2019). Angioedema in stroke patients with thrombolysis. Stroke.

[11] Hill MD, Buchan A, Canadian Alteplase for Stroke Effectiveness Study (CASES) Investigators (2005). Thrombolysis for acute ischemic stroke: results of the Canadian Alteplase for Stroke Effectiveness Study. CMAJ.

[12] Pahs L, Droege C, Kneale H (2016). A novel approach to the treatment of orolingual angioedema after tissue plasminogen activator administration. Ann Emerg Med.

[13] Powers WJ, Rabinstein AA, Ackerson T (2018). 2018 Guidelines for the Early Management of Patients with Acute Ischemic Stroke: A Guideline for Healthcare Professionals from the American Heart Association/American Stroke Association. Stroke.

